# Postoperative adjuvant chemoradiotherapy versus postoperative adjuvant radiotherapy for head and neck squamous cell carcinoma with adverse pathology: a systematic review and meta-analysis^☆^

**DOI:** 10.1016/j.bjorl.2024.101516

**Published:** 2024-10-04

**Authors:** Gabriela Garcia Korczaguin, Gilberto Vaz Teixeira, Ashok Shaha

**Affiliations:** a Universidade Federal de Santa Catarina, Florianópolis, SC, Brazil; b Centro de Pesquisas Oncológicas (CEPON), Serviço de Cabeça e Pescoço, Florianópolis, SC, Brazil; c Memorial Sloan-Kettering Cancer Center, Head and Neck Service, New York, USA; d Cornell University Medical College, New York, USA

**Keywords:** Head and neck squamous cell carcinoma, Adjuvant therapy, Chemoradiotherapy, Radiotherapy, Meta-analysis

## Abstract

**Objectives:**

Postoperative chemoradiotherapy has arisen as an adjuvant option for head and neck cancers, but its superiority to radiotherapy alone in patients with adverse pathologic factors is not yet well defined. We aimed to perform an updated meta-analysis comparing outcomes in head and neck cancer patients with adverse pathologic factors who underwent postoperative chemoradiotherapy and radiotherapy alone.

**Methods:**

We systematically searched PubMed, Scopus and the Cochrane Central Register of Controlled Trials for Randomised Controlled Trials (RCTs) in patients submitted to postoperative adjuvant therapy with radiotherapy alone or chemoradiotherapy.

**Results:**

We included 8 studies with a total of 2376 patients, of whom 1183 (49.8%) underwent postoperative chemoradiotherapy. In pooled analysis, overall survival (HR = 0.86; 95% CI 0.76‒0.98; *p* = 0.64; I^2^ = 0%) and disease-free survival (HR = 0.85; 95% CI 0.75‒0.96; *p* = 0.64; I^2^ = 0%) were shown to be superior in patients undergoing combined therapy. Chemoradiotherapy was also associated with significantly lower locoregional recurrence. However, there was no significant difference in distant metastasis occurrence between both groups. In an analysis of the extracapsular extension subgroup, the overall survival (OR = 3.12; 95% CI 1.76–5.51; *p* = 0.78; I^2^ = 0%), disease-free survival (OR = 3.44; 95% CI 2.00‒5.91; *p* = 0.68; I^2^ = 0%), and locoregional control (OR = 1.86; 95% CI 1.16‒2.99; *p* = 0.98; I^2^ = 0%) were better in the postoperative adjuvant chemoradiotherapy branch over radiotherapy alone.

**Conclusion:**

The results of our meta-analysis suggest that postoperative adjuvant chemoradiotherapy in patients with head and neck cancer with adverse pathologic factors favors a superior survival and a better locoregional control compared to postoperative radiotherapy alone, despite not affecting the ocurrence of distant metastasis.

## Highlights

• Overall and disease-free survival are greater in the adjuvant chemoradiation group.
• Head and neck cancer recurrence is lower in the adjuvant chemoradiation group.
• The rate of metastasis does not significantly differ between the adjuvant groups.
• The rate of late toxicity does not significantly differ between the adjuvant groups.
• The rate of acute skin toxicity is lower in the adjuvant chemoradiation group.

## Introduction

Head and neck cancers comprise a series of malignancies arising from head and neck sites, including the oral cavity, pharynx, larynx, and maxillary sinuses. Even though the advancements in diagnosis and treatments contributed to a better understanding and control of this group of malignancies, it still is a significant cause of worlwide mortality and morbidity.^1^ The development and the refinement of postoperative adjuvant therapeutic options have been crucial to the improvement of survival and locoregional control of these tumors, remarkably in patients with adverse pathologic factors that predispose individuals to a worse prognosis.^2^

Radiotherapy as adjuvant therapy has shown to provide effective locoregional control and survival improvement in patients submitted to advanced head and neck cancer resection. However, the emergence of the combination of postoperative chemotherapy and radiation as an option has introduced a new perspective and raised new questions in the adjuvant setting, regarding both the macroscopic and the microscopic disease.^3^

In spite of the propitious outcomes related to the combined therapy, the best therapeutic strategy for head and neck cancer patients with specific adverse pathologic features persists a subject to be studied and discussed. Various trials, randomized and observational, have assessed the advantages and safety aspects linked to both postoperative adjuvant chemoradiotherapy and radiotherapy alone in this population, yielding promising results favoring the combined therapy regimen.^4^ Most of the studies analyzed factors regarding the primary tumor, but few have reported outcomes related to the nodal lymphatic disease. Therefore, a comprehensive and systematic analysis of the available data is key to a better understanding of the actual benefits and risks of each therapy arm in this population.

Bearing in mind these contemplations, the present systematic review and meta-analysis aims to assess the benefits and risks of combining chemotherapy and radiation as an adjuvant therapy option for patients with resected head and neck squamous cell carcinoma with adverse pathologic factors. This study wants to provide embasement for clinical decisions and improvements of treatment and control of this group of malignancies.

## Methods

### Eligibility criteria

To be included in this meta-analysis, the studies had to meet all of the following eligibility criteria: (1) randomized trials; (2) enrolling patients with head and neck squamous cell carcinoma with at least one adverse pathologic feature who underwent resection of the primary tumor; (3) comparing postoperative chemoradiotherapy to postoperative radiotherapy alone. Adverse pathological features were: positive margins, close margins, pT3 or pT4 primary, perineural invasion, vascular invasion, lymphatic invasion, pN2 or pN3 nodal disease, nodal disease in levels IV or V and extranodal extension. Furthermore, to be included, each study had to report at least one of the main clinical outcomes of interest: overall survival, overall deaths or disease-free survival. Exclusion criteria were: (1) previous systemic therapy for any cancer, (2) distant metastasis at the time of diagnosis, (3) no control group, (4) inadequate intervention group. Follow-up was not considered a major criterium for inclusion/exclusion of studies.

Laramore et al.^11^ included 448 patients with Stage III and IV cancers, and although the study also included Stage II hypopharyngeal cancers, the majority were Stage III and IV. The inclusion of Stage II hypopharyngeal cancers was deemed acceptable as the overall patient population met the meta-analysis criteria, focusing primarily on high-risk features and advanced stages. Thus, all 448 patients were included.

Stage III and IV cancers, while not explicitly mentioned in the criteria, were included due to their alignment with the adverse pathological features central to our analysis. These stages often involve significant adverse features such as large primary tumors (pT3 or pT4), extensive nodal involvement (pN2 or pN3), and positive margins. Including these stages ensures a comprehensive evaluation of postoperative chemoradiotherapy versus radiotherapy alone, reflecting high-risk scenarios and providing a robust analysis applicable to the target patient population.

### Search strategy and data extraction

The following databases were systematically searched from inception to December 2023: PubMed, Scopus, and Cochrane Central Register of Controlled Trials. The systematic search strategy was: (“head and neck”) AND (“squamous cell carcinoma” OR “squamous-cell carcinoma” OR cancer OR carcinoma OR hnscc) AND (adjuvant OR postoperative) AND (chemoradiation OR chemoradiotherapy OR pocrt OR crt OR chemotherapy) AND (radiotherapy OR radiation OR irradiation OR port OR rt) AND (extracapsular OR extranodal OR advanced OR “high-risk”) AND (randomized OR randomised). In order to find additional relevant studies and validate the search strategy, the references from the included studies and previous systematic reviews and meta-analyses were manually reviewed through snowballing.

### Endpoints

Overall survival, overall number of deaths, and disease-free survival were the main outcomes of interest. All outcomes included overall survival, overall number of deaths, disease-free survival, locoregional recurrence, distant metastasis, late toxicity, and local severe acute skin adverse events — at least Grade 3.

### Quality assessment

The risk of bias of all studies was evaluated through RoB-2 by the independent authors. Disagreements were resolved through a consensus between the authors.

### Statistical analysis

Publication bias was assessed using funnel-plot analysis of point estimates in relation to study weights. This systematic review and meta-analysis was performed and reported in accordance with the Cochrane Collaboration Handbook for Systematic Review of Interventions and the Preferred Reporting Items for Systematic Reviews and Meta-Analysis (PRISMA) statement guidelines. Hazard-Ratios (HR) with 95% Confidence Intervals were used to compare therapies effects for survival endpoints. Odds-Ratios (OR) with 95% Confidence Intervals were used to compared therapies effects for categorical endpoints. Heterogeneity was assessed with I^2^ statistics and Cochrane Q test; *p*-values < 0.10 and I^2^ > 25% were considered significant for heterogeneity. We used a DerSimonian and Laird random effect model for all outcomes. Review Manager 5.4.1 (Cochrane Center, The Cochrane Collaboration, Denmark) was used for statistical analysis.

### Study selection and baseline characteristics

**Figure 1:**
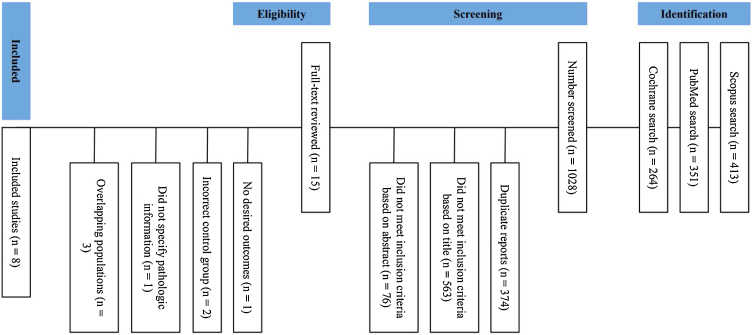
PRISMA flow diagram of study screening and selection.

The initial search, reported in Fig. 1, yielded 1028 studies. 15 studies were selected for full reading after removal of duplicate records and studies that did not meet the inclusion criteria based on title and abstract. Among these 15 studies, 8 randomized controlled trials with a total of 2376 patients were included. 1183 (49.8%) patients were randomized to undergo postoperative adjuvant chemoradiotherapy, of whom 189 (16%) received Carboplatin, 712 (60%) received Cisplatin only, 223 (19%) received Cisplatin and 5-Fluorouracil, and 59 (5%) received Mitomycin C and Bleomycin in addition to radiation. Study characteristics are detailed in Table 1. Most of the patients were men and no older than 65 years. Median, mean or minimum follow-up was equal to or greater than 60 months in 7 studies. One study reported a mean follow-up of 45.7 months. The median follow-up of the preliminary reports of Bachaud et al.,^6^ and Cooper et al.,^9^ were 36 and 45.9 months, respectively. The primary tumor sites for the postoperative radiotherapy and postoperative chemoradiotherapy arms were, respectively: 28% (n = 209) and 22% (n = 164) oral cavity; 28% (n = 207) and 35% (n = 253) oropharynx; 18% (n = 130) and 16% (n = 120) hypopharynx; 9% (n = 69) and 11% (n = 80) larynx. Since not all studies provided information on primary tumor sites, this analysis was performed considering the ones that did provide these data, representing a sample of 1467 patients, and 909 patients had their primary tumor sites grouped as head and neck squamous cell cancer.

**Table 1:** Baseline characteristics of included studies.

Study	Country	Study period	Patients (RT/CRT)	Male (%) (RT/CRT)	Median age ≤65 years	T3, T4 or IT (%) (RT/CRT)	N2 or N3 (%) (RT/CRT)
Argiris et al.^5^	USA	04/1994–04/2002	36/36	75/92	Yes	N/A	92/81
Bachaud et al.^7^	France	04/1984–04/1988	44/39	N/A	Yes^a^	N/A	N/A
Bernier et al.^8^	Switzerland	02/1994–10/2000	167/167	93/92	Yes	63/69	57/57
Cooper et al.^10^	USA	09/1995–04/2000	210/206	86/86	Yes^a^	N/A	N/A
Laramore et al.^11^	USA	01/1985–01/1990	225/223	84/83	N/A	73/72	42/37
Laskar et al.^12^	India	06/2005–03/2013	299/300	87/87	Yes	10.7/10.3	50.5/52
Porceddu et al.^13^	Australia	04/2005–07/2014	157/153	94/92	Yes	N/A	N/A
Zakotnik et al.^15^	Slovenia	03/1997–12/2001	55/59^b^	89/90^b^	Yes^b^	N/A	N/A

Study	Positive SM (%) (RT/CRT)	LVI and/or LVE (%) (RT/CRT)	ECE (%) (RT/CRT)	Poor diff. (%) (RT/CRT)	PNI (%) (RT/CRT)	RT delay > 6 w (RT/CRT)	RT delay > 100 d (RT/CRT)
Argiris et al.^5^	28/30	17/33	78/72	14/17	19/19	N/A	N/A
Bachaud et al.^7^	40.91/28.21^a^	N/A	100/100	9.09/7.69^a^	N/A	14/13^a^	N/A
Bernier et al.^8^	26/31	19/21	53/61	19/18	14/13	25/32	N/A
Cooper et al.^10^	19/17^a^	N/A	N/A	7/7^a^	N/A	N/A	N/A
Laramore et al.^11^	N/A	N/A	N/A	N/A	N/A	N/A	N/A
Laskar et al.^12^	0.3/0.7	3.3/3.7	55.9/53.3	N/A	28.4/24.7	N/A	10.7/12.7
Porceddu et al.^13^	10.8/18.3	N/A	60/58	N/A	N/A	11/12	N/A
Zakotnik et al.^15^	N/A	22/15^b^	51/54^b^	N/A	7/2^b^	N/A	N/A

Study	RT regimen	CT regimen	CT drug	Follow up (mo)
Argiris et al.^5^	1.8 Gy 5×/w (total of at least 59.4 Gy over 6.5 w)	1 w, prior to RT, for 6 w during RT	Carboplatin	63.6 (median)
Bachaud et al.^7^	1.7 Gy 5×/w for the first 54 Gy + 1.8–2 Gy/day until completion	50 mg, IV, on the first day of each week of irradiation course	Cisplatin	60 (minimum)
Bernier et al.^8^	2 cGy 5×/w over a 5.5 w period ± boost	100 mg/m^2^ on days 1, 22, and 43 of radiotherapy	Cisplatin	60 (median)
Cooper et al.^10^	60 Gy in 30 fractions over a 6 w period ± boost	101 mg/sq m of body surface on days 1, 22, and 43 of radiotherapy	Cisplatin	112.8 (median)
Laramore et al.^11^	1.8‒2 cGy 5×/w (High-risk: 60 Gy)	Cisplatin 100 mg/m^2^ on day 1 + 5-FU at 1 g/m^2^ on days 1–5; 3 cycles repeated at 21-day intervals	Cisplatin and 5-FU	45.7 (mean)
Laskar et al.^12^	60 Gy in 30 fractions, 5×/w, over a 6 w period	Weekly I.V. 30 mg/m^2^ for 5–6 w	Cisplatin	93.5 (median)
Porceddu et al.^13^	60 Gy in 30 fractions over a 6 w period (66 Gy if positive margins)	Weekly Carboplatin dose (Calvert formula) for 6 weeks (maximum)	Carboplatin	60 (median)
Zakotnik et al.^15^	CF 2 cGy 5×/w (56–70 Gy)	Mitomycin C at 15 mg/m^2^ after 10 Gy; 5 mg of Bleomycin I.M. 2×/w during irradiation	Mitomycin C and Bleomycin	76 (median)

RT, radiotherapy; CRT, chemoradiotherapy; USA, United States of America; IT, in transit; SM, surgical margins; PNI, perineural invasion; LVI, lymphovascular invasion; LVE, lymphovascular embolism; diff., differentiated; mo, months; w, weeks; d, days; I.V., intravenous; I.M., intramuscular. 5-FU, 5-fluorouracil.

a Data reported in the respective preliminary results of the study.

b Same population as Šmid et al.^14^

## Results

### Pooled analysis of studies

**Figure 2:**
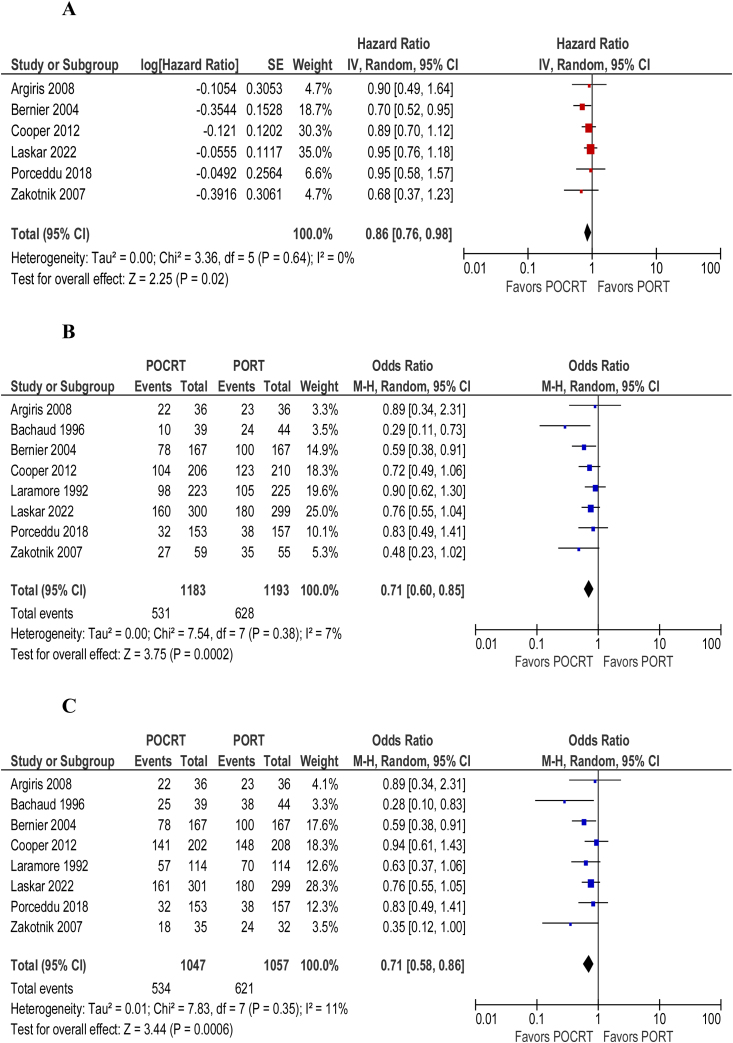
(A) Forest plot of overall survival. (B) Forest plot of disease-free survival. (C) Overall number of deaths.

**Figure 3:**
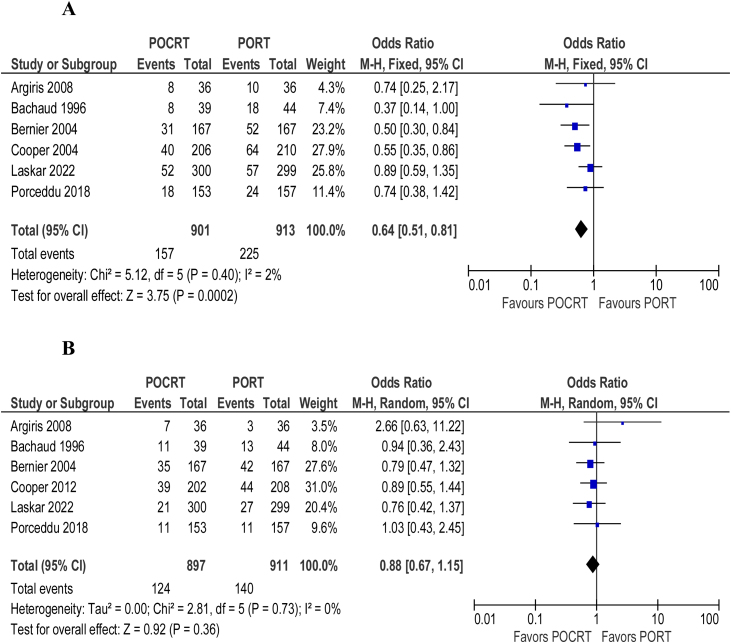
(A) Forest plot of locoregional recurrence. (B) Forest plot of distant metastasis.

**Figure 4:**
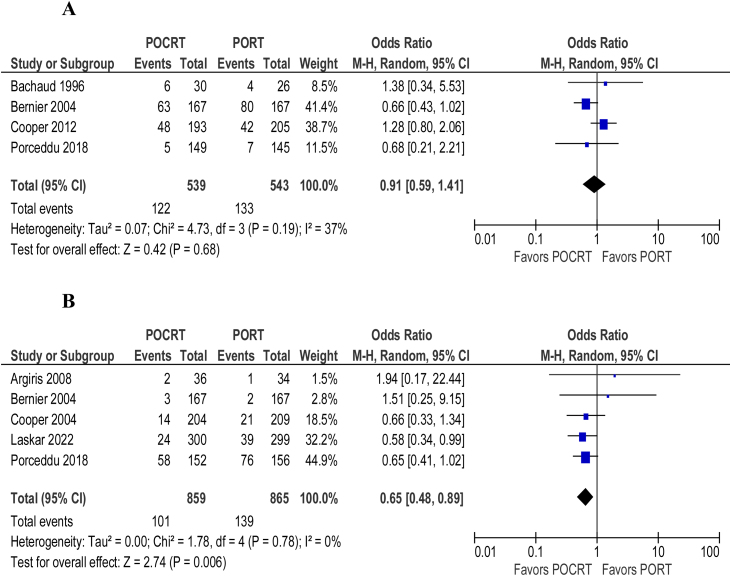
(A) Forest plot of late toxicity. (B) Forest plot of severe local acute skin adverse events.

Overall survival (HR = 0.86; 95% CI 0.76‒0.98; *p* = 0.64; I^2^ = 0%) (Fig. 2) and disease/progression-free survival (HR = 0.85; 95% CI 0.75‒0.96; *p* = 0.64; I^2^ = 0%) (Fig. 2) were shown to be better in patients undergoing adjuvant chemoradiotherapy over adjuvant radiotherapy alone. The overall number of deaths (OR = 0.71; 95% CI 0.58‒0.86; *p* = 0.35; I^2^ = 11%) (Fig. 2) and locoregional recurrence (OR = 0.66; 95% CI 0.53‒0.82; *p* = 0.5; I^2^ = 0%) (Fig. 3) were significantly lower in patients undergoing adjuvant chemoradiotherapy. The ocurrence of distant metastasis (OR = 0.88; 95% CI 0.67‒1.15; *p* = 0.73; I^2^ = 0%), on the other hand, did not significantly differ between adjuvant radiotherapy alone and adjuvant chemoradiotherapy (Fig. 3). In an analysis of adverse events and toxicity, adjuvant chemoradiotherapy was associated with a lower ocurrence of local acute skin adverse events (OR = 0.65; 95% CI 0.48‒0.89; *p* = 0.78; I^2^ = 0%) (Fig. 4). The ocurrence of late toxicity (OR = 0.91; 95% CI 0.59–1.41; *p* = 0.19; I^2^ = 37%) (Fig. 4) was not shown to be significantly different between both adjuvant options.

### Subgroups

**Figure 5:**
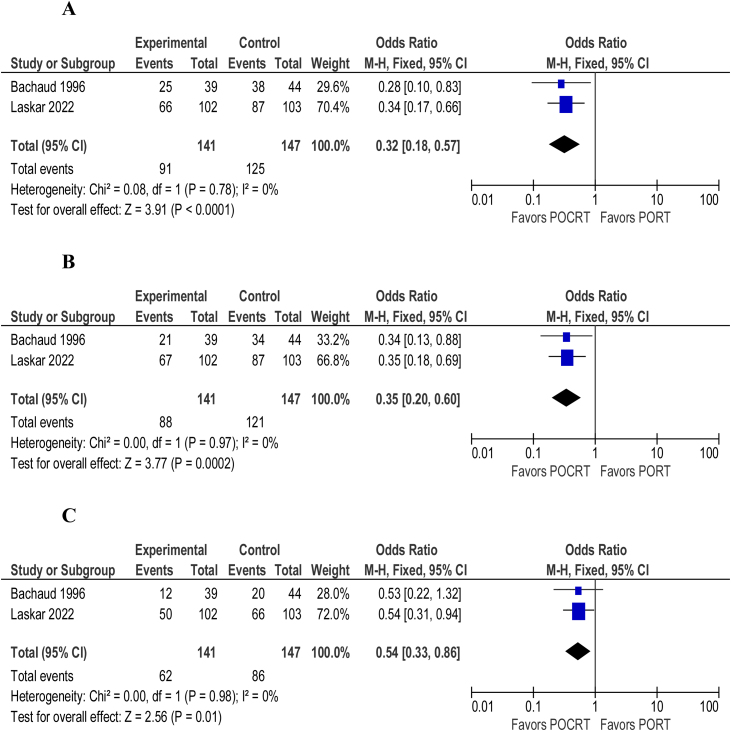
(A) Forest plot of overall number of deaths — extracapsular extension subgroup. (B) Forest plot of disease recurrence — extracapsular extension subgroup. (C) Forest plot of locoregional recurrence — extracapsular extension subgroup.

Overall survival (OR = 3.12; 95% CI 1.76–5.51; *p* = 0.78; I^2^ = 0%), disease-free survival (OR = 3.44; 95% CI 2.00–5.91; *p* = 0.68; I^2^ = 0%) and locoregional control (OR = 1.86; 95% CI 1.16–2.99; *p* = 0.98; I^2^ = 0%) of patients with lymph node extracapsular extension on postoperative anatomopathologic final reports were analyzed, significantly favoring the chemoradiotherapy branch (Fig. 5).

### Quality assessment

**Figure 6:**
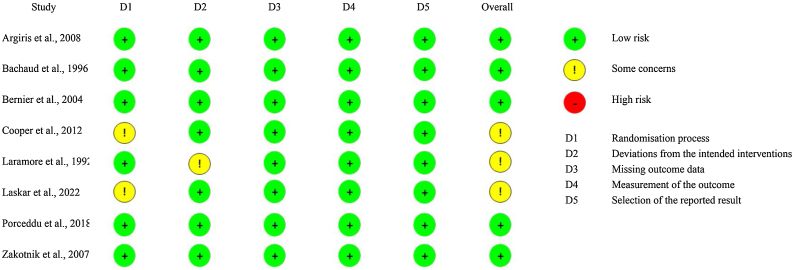
Bias assessement.

Fig. 6 reports the individual appraisal of each study included. Overall, the studies were classified at low risk of bias. The funnel plot analyses of the main outcomes (overall survival and overall number of deaths) did not show evidence of publication bias.

## Discussion

In this systematic review and meta-analysis of 8 studies and 2109 patients, we compared postoperative adjuvant chemoradiotherapy versus postoperative adjuvant radiotherapy for head and neck squamous cell carcinoma with adverse pathologic factors. The main findings were: (1) overall survival and disease-free survival were significantly better in patients undergoing chemoradiotherapy;^19^ (2) although locoregional recurrence was lower in chemoradiotherapy, the ocurrence of distant metastasis was not significantly different between both adjuvant options; (3) in spite of the small number of studies reporting outcomes for the extracapsular spread subgroup individually, the overall survival, disease-free survival, and locoregional control are suggested to be superior in patients submitted to postoperative adjuvant chemoradiotherapy; (4) the occurrence of late adverse events was not significantly different between adjuvant chemoradiotherapy and adjuvant radiotherapy alone, but local acute skin adverse events were significantly more common in the radiotherapy groups.

The National Comprehensive Cancer Network Guidelines for Head and Neck Cancer recommends postoperative adjuvant treatment for patients with head and neck cancer with adverse pathologic features. Even though other meta-analyses^16^, ^17^, ^18^ have already compared postoperative adjuvant chemoradiotherapy versus postoperative adjuvant radiotherapy alone in order to find out the best adjuvant therapy option, our updated systematic review and meta-analysis included only patients with the adverse pathologic features described by NCCN (2023), so as to identify more precisely the impact of both therapies in these patients. The benefits of postoperative adjuvant chemoradiotherapy over radiotherapy, on overall and disease-free survivals were described by Shang et al.^16^ and Winquist et al.^17^ in 2014 and 2017 respectively. However, Shang et al. included not only randomized controlled trials, but one retrospective cohort as well. Winquist et al.’s meta-analysis, despite being more recent and including final reports of trials, did not report outcomes other than overall survival and locoregional control. A previous systematic review and meta-analysis performed by Winquist et al.^18^ in 2007 also reported a statistically significant benefit in overall survival and a lower overall number of deaths of adjuvant chemoradiotherapy compared to adjuvant radiotherapy alone. Nonetheless, this review included a small number of studies and didn’t include the final reports of many trials that were included in our meta-analysis.

The findings of our systematic review and meta-analysis underline the cardinal role of postoperative adjuvant chemoradiotherapy as a systemic therapy option for head and neck squamous cell carcinoma with adverse pathologic features. The association of radiation and chemotherapy may enhance locoregional control and attenuate the risk of tumor recurrence. Even though these aspects are consistent with previous meta-analyses, our updated assessment targeted patients with the adverse pathologic features defined by the NCCN (2023). Remarkably, while chemoradiotherapy provided a significant reduction in locoregional recurrence, it has not shown significant benefit in the control of distant metastases, warranting further investigation with randomized controlled trials.

Despite the insights reported in our meta-analysis suggesting various benefits from the addition of chemotherapy to the postoperative irradiation of head and neck squamous cell carcinoma patients, it is important to acknowledge some limitations. The scarcity of individual patient data in some trials regarding specific pathologic features might have limited the ability to analyze subgroups and address the association of each treatment arm on each individual pathologic factor. Although it was possible to analyze the extracapsular spread subgroup, there were a limited number of studies that matched the required features for this analysis. The shortage of patient detailing about late adverse events limited the assessment of system-specific events as well.

Regarding the extracapsular spread subgroup, there is significant suggestion of the benefit of postoperative adjuvant chemoradiotherapy over postoperative adjuvant radiotherapy alone in overall survival, disease-free survival, and locoregional control of the disease, indicated by the significantly lower overall number of deaths, disease recurrence, and locoregional recurrence found. These findings point that extracapsular extension may correspond to an important prognostic factor. Most available studies have analyzed tumor characteristics, but few studies have investigated the impact of nodal disease in the prognosis of patients subjected to surgery. Our findings reported a significant difference between the outcomes arising from head and neck cancer patients with extracapsular nodal extension who underwent surgery, favoring the postoperative chemoradiotherapy branch as the best adjuvant option.

In the analyses of adverse events, the analogous occurrence of late adverse events in both chemoradiotherapy and radiotherapy alone suggests that adding chemotherapy to postoperative radiation does not confer the patient a worse prognosis related to late toxicity. On the other hand, the significantly higher rates of local acute skin adverse events in patients subjected to postoperative radiotherapy alone may highlight a previous undescribed advantage of chemoradiotherapy over radiotherapy.

Our study yielded significant evidence indicating several benefits of postoperative adjuvant chemoradiotherapy over radiotherapy alone in head and neck squamous cell carcinoma patients presenting adverse pathologic features. Analogue findings were reported by the pivotal studies published in New England Journal of Medicine by Bernier et al. and Cooper et al. The first, a randomized controlled trial, has provided vital understanding regarding the efficacy of adjuvant combined therapy – chemoradiotherapy – and settled foundation for further research into the particularities of each therapy and their prognostic features for patients with head and neck cancer with adverse pathologic features. Despite not indicating a benefit in overall survival of the population in any of the reports (preliminary and final), the locoregional control was found to be statistically significantly better in patients submitted to the combined modality of treatment in both reports. Similarly, there was a statistically significant greater rate of acute adverse events in the chemoradiotherapy group. However, in a separate analysis of local skin adverse events, the results showed a reduced incidence of these events in the chemoradiotherapy arm, data that certainly contributed to the unexpected finding reported in the present meta-analysis of a significant benefit of combined therapy over radiotherapy alone regarding local acute skin adverse events. Regarding the disease-free survival rates, the benefit of the combined therapy was significant in the preliminary report, but lost the significance in the final report after a 10-year follow-up for the general population. When studying subgroups, the benefit was maintained in patients with extracapsular extension and/or positive margins, but it was not identified in patients with multiple lymph nodes only, what suggests that multiple lymph node compromising is not an important prognostic factor regarding treatment response for radiotherapy alone or combined modality. The rates of late adverse events, in both reports, similarly to the results of the present meta-analysis, were not remarkably distinct between the groups.

We expected a significant difference in the distant metastasis on the chemoradiotherapy arm based on systemic treatment, but this was not observed in this meta-analysis. It may be due to other factors that were not well-defined.

The analysis reveals that adjuvant chemoradiotherapy (POCRT) with cisplatin significantly reduces locoregional recurrence in head and neck cancer patients compared to radiotherapy alone. However, it shows no significant advantage in reducing distant metastasis. Cisplatin appears more effective than carboplatin in both outcomes, but neither significantly increases late toxicity, with a slight advantage of cisplatin in reducing acute skin toxicity. These findings suggest that cisplatin should be preferred in POCRT for its enhanced efficacy and manageable toxicity profile.

Bernier’s study, by investigating the role of postoperative irradiation with or without concomitant chemotherapy in locally advanced head and neck cancer, reported relevant improvement of locoregional control, overall survival, and progression-free survival (that was included in the disease-free survival analysis in the present meta-analysis) when chemotherapy was associated to radiotherapy as the adjuvant therapy for head and neck cancer patients with adverse pathologic features that underwent surgery previously. Furthermore, severe adverse events were found to be statistically significantly greater in the chemoradiotherapy group — including the local acute skin adverse events, in opposition to Cooper’s study, but the late adverse events did not show an important difference between the branches of the study. Thereby, the survival and locoregional control rates reported in Bernier’s study are aligned with the present study findings.

It is noticeable that the incorporation of chemotherapy in the adjuvant irradiation therapy is beneficial to the patients regarding their survival and locoregional control. However, it is imperative to address the toxicity profile of this association, especially concerning the medications which are commonly used as chemotherapeutic agents (cisplatin, carboplatin, 5-fluorouracil, and others), and their broad spectrum of adverse effects that influence the patient’s life quality and treatment adherence. These toxicities encircle acute and late adverse events, and may affect different body systems and organs. Some of the most observed acute events include nausea, vomiting, mucositis, and myelosuppression, which can seriously deteriorate the patient’s health status and require interventions. On the other hand, the late toxicity deriving from chemotherapeutic agents comprises effects ranging from peripheral neuropathy to secondary malignancies and multi-organs impairment. However, unlikely some of the acute adverse events, few studies have precisely identified the impact of adding chemotherapy to the adjuvant irradiation for post-surgical head and neck cancer patients with adverse pathologic features compared to the long-term effects of adjuvant irradiation alone, as happened in the present study due to a statistically insignificant outcome. Thereby, it is crucial developing strategies to mitigate treatment-related toxicity — both acute and late, so as to allow the completion of the treatment with minimal unforeseen events, interruptions or health losses, and to give the best possible quality of life for the patient.

## Conclusion

Our updated systematic review and meta-analysis reported unassailable evidence indicating the superiority of postoperative adjuvant chemoradiotherapy over radiotherapy alone in the survival and locoregional disease control, as well as local acute skin toxicity, in patients with head and neck squamous cell carcinoma of the head and neck with adverse pathologic features according to NCCN 2023.

## Ethics committee approval

This meta-analysis was exempt from requiring approval from an ethics committee because it involved the synthesis of data from previously published studies and did not directly involve human subjects.

The authors states that the work in appreciation has not been published previously and it is not under consideration for publication elsewhere.

## Funding

This research did not receive any specific grant from funding agencies in the public, commercial, or not-for-profit sectors.

## Conflicts of interest

The authors declare no conflicts of interest.
